# Success rate of inferior alveolar nerve block in diabetic patients with symptomatic irreversible pulpitis: a prospective study

**DOI:** 10.1186/s12903-025-07312-4

**Published:** 2025-11-22

**Authors:** Rakesh Singla, Mani Gera, Gurdeep Singh Gill, Namita Jain, Adel Saeed N. Alobaid, Suraj Arora, Ahmed Abdullah Al Malwi, Shan Sainudeen, Priyanka Saluja, Waled Abdulmalek Alanesi

**Affiliations:** 1 Department of Conservative Dentistry and Endodontics, Jan Nayak Chaudhary Devilal Dental College, Haryana Sirsa, India; 2 Department of Restorative Dental Sciences, College of Dentistry, King Khalid University, Abha, Saudi Arabia; 3 Mike Petryk School of Dentsitry, University of Alberta, Edmonton, AB Canada; 4 Department of Operative Dentistry, Faculty of Dentistry, University of Science and Technology, Inmaa City, Aden, Yemen

**Keywords:** Diabetes mellitus, Inferior alveolar nerve block, Local anesthesia, Symptomatic irreversible pulpitis

## Abstract

**Background:**

The global prevalence of Diabetes Mellitus is steadily increasing, leading to a larger diabetic population undergoing root canal treatment (RCT). However, clinical studies assessing anesthetic success in diabetic patients are lacking. In diabetic patients, local anesthetic success may be affected owing to neurovasular changes in dental pulps. This study is intended to establish baseline data regarding local anesthetic success in the diabetic population undergoing RCT. The study’s main purpose was to assess the success rate of Inferior Alveolar Nerve Block (IANB) in type 2 diabetic patients during RCT of mandibular molars with symptomatic irreversible pulpitis (SIP).

**Methods:**

Sixty-five adult patients diagnosed with SIP in mandibular molars were divided into 2 groups according to their glycated hemoglobin (HbA1c) levels; control group (*n* = 31, HbA1c < 5.7) and diabetic group (*n* = 34, HbA1c levels > 6.5). After administering IANB and achieving lip numbness, endodontic access was initiated. Pain was recorded during endodontic procedure using Heft Parker visual analogue scale. Anesthetic success was defined as no or mild pain [score 0–54 mm] throughout endodontic procedure. Chi square test and Mann Whitney U tests were used for statistical analysis.

**Results:**

Patients in the diabetic group exhibited significantly higher anesthetic success (94%) as compared to non-diabetic group (74%) (*P* = 0.016). Patients in the control group were significantly younger than the diabetic group (*P* < 0.001). Logistic regression revealed that tooth number was significantly associated with anesthetic success while age and gender had no significant influence on anesthetic success.

**Conclusions:**

Within the study limitations, IANB appeared more successful in diabetic patients compared to non-diabetics during RCT of mandibular molars with SIP. Studying local anesthetic success in diabetics is vital for enhancing patient comfort, safety and optimizing outcomes during RCT.

**Trial registration:**

The clinical trial was registered on 24th September 2021 and registration no. is CTRI/2021/09/036813.

## Background

Diabetes mellitus (DM) is a clinically and genetically heterogenous group of disorders affecting the metabolism of carbohydrates, lipids, and proteins in which hyperglycemia is the main feature [1]. Type 2 DM accounts for 90–95% of DM cases and requires insulin and/or other drugs to control blood glucose levels [2]. India has an estimated 77 million people with type 2 DM, making it the second most affected country in the world after China [3]. Among medically compromised patients, diabetics are the third most prevalent group seeking dental treatment [4]. The number of diabetic patients globally is projected to grow to 700 million by 2045 [3]. With such staggering growth in the number of diabetic patients and increasing awareness about tooth preservation, more diabetic patients are set to undergo root canal treatment (RCT).

Dental pulps of diabetic patients may differ from those of healthy patients. Studies [5, 6] have demonstrated that the dental pulp vasculature may be markedly affected by diabetic status of an individual. Long-standing DM resulted in angiopathy, pulpal fibrosis and a thickened basement membrane in pulpal blood vessels [5, 6]. Dental pulps of streptozotocin-induced diabetic rats demonstrated high expression of inflammatory mediators, such as kallekrien, and enzymes, including myeloperoxidases and alkaline phosphatases [7]. In a recent laboratory study [8], clinically normal pulps isolated from well-controlled type 2 diabetic individuals exhibited greater expression of inflammatory cytokines, including IL-1β, IL-6, IL-17, and TNF-α.

Inferior Alveolar Nerve Block (IANB) is the most preferred technique for RCT of mandibular molars but has a high failure rate in patients with irreversible pulpitis (IP), primarily due to pulpal inflammation [9]. In diabetic patients suffering from IP, it can be assumed that existing pulpal inflammation, coupled with increased expression of inflammatory mediators and enzymes, may affect anesthetic success. However, data regarding IANB success in such patients is lacking as there are no endodontic studies performed exclusively on diabetic population. Most of the previously conducted anesthetic evaluation studies [10, 11] have included systemically healthy patients [American Society of Anesthesiologists (ASA I]. Few studies included patients with controlled diseases (ASA II) as well but did not provide separate data on diabetic patients [9]. In a recently published research network study [12], diabetic patients undergoing non-surgical endodontic treatment had almost 5 times higher chances of anesthesia failures. However, in that survey, the diabetic status of patients was self-reported, and the type of local anesthesia(LA) was not specified.

Establishing baseline data on the effectiveness of local anesthetics in diabetic patients is vital for refining anesthesia protocols. By analyzing this information, dental professionals can enhance patient outcomes by reducing intraoperative pain and complications. Additionally, this effort will support the development of evidence-based guidelines tailored to the unique physiological needs of diabetic individuals, ultimately improving the safety and effectiveness of anesthetic management during endodontic procedures.

Therefore, this study aimed to evaluate the success rate of IANB among type 2 diabetic patients, during RCT in mandibular molars with symptomatic IP (SIP). The null hypothesis for the study was that there would be no significant difference in the success rate of IANB between type 2 diabetic and non-diabetic patients during RCT of mandibular molars with SIP.

## Materials and methods

This single-centre, prospective clinical study was conducted between January 2021 and July 2022 in the Department of Endodontics, following ethical clearance from the Institutional Ethics Committee. The study was registered prospectively on the Clinical Trials Registry, and written informed consent was obtained from all study participants.

### Sample size calculation

Sample size was estimated using nMaster software (version 2, CMC, Vellore). The sample size was estimated for “Hypothesis testing of two large proportions: Equal allocation”. Based on pilot study results, it was anticipated that the success rate of IANB among diabetic patients as 94.5% and among non-diabetics as 65%, with a power of 80% and a 95% confidence interval, a sample size of 56 [28 per group] was found to be sufficient. Considering 10% dropouts, the total sample size was inflated to be 62 i.e. 31 per group. Patients were recruited in the study until the desired sample size was reached.

#### Inclusion criteria

Consented patients ageing 18 to 72 years complaining of moderate to severe pain [> 54 mm on Heft Parker visual Analogue scale (HP-VAS)] [13] in one of their mandibular molars diagnosed with SIP and with normal periapical radiographic appearance (Periapical Index Score ≤ 2) [14] were included in the study. The diagnosis of SIP was established based on the patient’s subjective symptoms (sharp, lingering, spontaneous or referred pain) and confirmed by an early and prolonged painful response to the cold test (Endo-Frost, Coltene, Germany). Patients who gave informed consent for an HbA1c test and a physician consultation for the diagnosis of diabetes (if HbA1c levels were above 6.5) were included in the study.

Patients having prediabetes (i.e. HbA1c levels from 5.7 to 6.4), any other type of diabetes, poorly controlled diabetes (HbA1c > 8), diabetic patients taking insulin, with associated systemic conditions like coronary heart disease, hypertension, hyperparathyroidism, liver or kidney diseases, smokers, pregnant or lactating mothers, patients with poor oral hygiene, bone loss, undergoing orthodontic treatment, patients who had taken antibiotics/analgesics within the last 24 h were excluded from the study. Pain assessment was performed using the HP-VAS. This scale consists of a 170-mm marked line divided into 4 sections, each with different terms describing levels of pain: No pain (0 mm), mild pain (1–74 mm), moderate pain (55–114 mm), and severe pain (115–170 mm). All patients were instructed and trained to use this scale before recording their pain levels.

### HbA1c sample collection and testing

The blood samples of all patients were collected by a trained phlebotomist and processed in the associated hospital laboratory for HbA1c levels. The results were obtained on the same day before RCT. Following American Diabetes Association (ADA) Criteria [2], Patients with HbA1c > 6.5 were included in Diabetic group, and patients having HbA1c levels < 5.7 were included in the non-diabetic (control) group. Patients with previously undiagnosed diabetes but HbA1c > 6.5 were sent for physician consultation to confirm diabetes before RCT.

Due to the nature of the study, randomisation of patients was not possible. The responsibility for patient screening was delegated to the senior lecturer, who was unrelated to the study. At all times, the operator was blinded to the patient’s glycemic status, which was revealed to the operator only at the end of the study. Being unaware of the patient’s diabetic status, the operator was instructed to take diabetes-related precautions for every patient [15].

### Clinical procedure

A single operator performed endodontic procedures. All patients received standard IANB with 1.8 mL cartridge of 2% lidocaine containing 1:200.000 epinephrine (Cadila Pharmaceuticals, Dholka, Gujarat,India) using a 27-G needle (Septoject, Septodont, rue du Pont de Créteil, Saint-Maur, France). Patients who did not report lip numbness within 15 min of LA administration were excluded. After confirming lip numbness, rubber dam was applied and endodontic access was initiated using Endo Access Kit (Dentsply Sirona Ballaigues, Switzerland); canal orifices were located with the DG 16 Endodontic Explorer (GDC Fine Crafted Dental Pvt. Ltd, Hoshiarpur, India), stainless steel K-files #06, 08, and 10 (Mani Inc. Utsunomiya, Tochigi, Japan) were used for the initial instrumentation of the root canals. Patients who did not report lip numbness or exhibited no haemorrhage from pulp chamber were excluded from the study. During endodontic procedure, patients were instructed to rate their pain on HP-VAS. No or mild pain (0–54 mm) during the procedure was counted as success, whereas moderate to severe pain (55–170 mm) at any stage (access opening or canal instrumentation) was considered as failure. The failure cases were treated in the same visits by another senior lecturer using supplemental blocks. Anesthetic related precautions and stress reduction protocol were followed in all patients. Before RCT, it was ensured that the patient had eaten normally before the treatment and had taken their medication (if diabetic). A blood glucose meter, glucose, and an insulin pen were kept on hand for use if necessary. If patient exhibited signs/symptoms of hypoglycemia during endodontic intervention, procedure was discontinued immediately, and standard hypoglycemic precautions were to be followed [15].

### Statistical analysis

Data was analyzed using Statistical package of Social Sciences [IBM version 16, Chicago, Illinois, USA] software. An independent t-test was used to compare mean age between the groups. Chi-square test was used for intergroup comparison of categorical variables (gender, tooth type). Since pain was recorded on an ordinal scale, a non-parametric test, i.e. Mann Whitney U test, was used for intergroup comparison of mean pain scores. Logistic regression analysis was used to assess the effect of age, gender and number of tooth on anesthetic success.

## Results

**Fig. 1:**
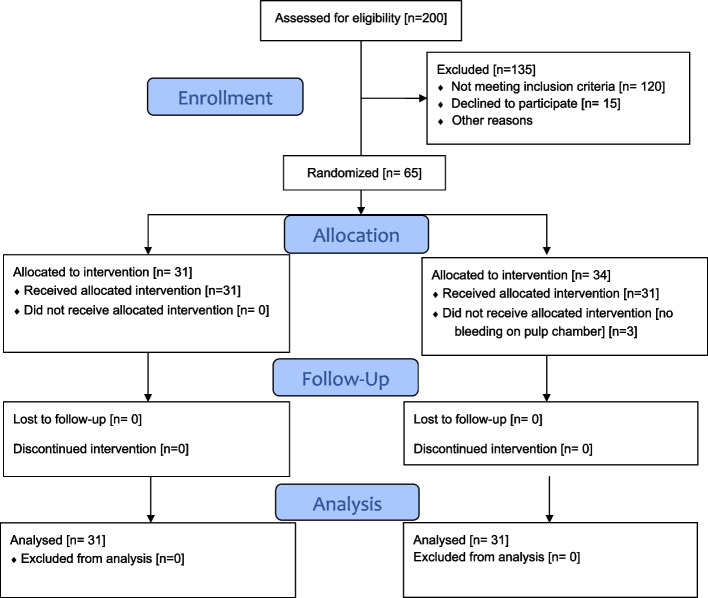
Flow diagram

A total of 65 patients (31 in the control group and 34 in the diabetic group) were recruited for the clinical trial. In the diabetic group, 3 patients were excluded as they did not exhibit haemorrhage from the pulp chamber. Finally, 62 patients (31 in each group) completed the clinical trial and were statistically analyzed (Fig. 1). The two groups had no significant differences regarding gender, tooth number, and preoperative pain scores. Patients in the control group were significantly younger than the diabetic group (*P* < 0.001) (Table 1). The success rate of IANB was significantly higher in the diabetic group than the control group (*p* = 0.016) (Table 2). Patients belonging to non-diabetic (control) group experienced significantly higher intraoperative pain as compared to diabetic group (*P* = 0.016) (Table 3). For regression analysis, age wise comparison was done by dividing patients into 2 categories: up to 40 year or more than 40-year. Logistic regression revealed that tooth number was significantly associated with anesthetic success (*P* = 0.02), while age (*P* = 0.24) and gender (*P* = 0.26) had no significant influence on anesthetic success (Table 4).

**Table 1:** Pre-operative variables

Variables	Control group	Diabetic group	*p*-value
Age [years]			
Mean & SD	33.12 & 10.77	57.15 & 9.35	< 0.001^*^
Gender n [%]			
Female	14 [45.2%]	18 [58.1%]	0.446
Male	17 [54.8%]	13 [41.9%]	
Tooth type n [%]			0.124
First molar	13 [41.9%]	21 [67.7%]	
Second molar	16 [51.6%]	9 [29.0%]	
Third molar	2 [6.5%]	1 [3.2%]	
Pre-operative pain			
Mean & SD	123.09 & 28.80	112.06 & 27.67	0.112

^*^Statistically significant

**Table 2:** Success rate of the Inferior alveolar nerve block

**Group**	**Success** [No pain to mild pain]	**Failure** [moderate to severe pain]	***p*-value**
Control	23 [74.2%]	8 [25.8%]	0.016^*^
Diabetic	29 [93.6%]	2 [6.5%]	

^*^Statistically significant

**Table 3:** Comparison of mean intraoperative pain scores (in mm) between groups

Group	Mean	Standard deviation	*p*-value
Control	33.87	36.87	0.016^*^
Diabetic	12.93	24.27	

^*^Statistically significant

**Table 4:** Logistic regression analysis

Logistic Regression Analysis with Success rate (intraoperative pain upto 54 mm) as dependent variable							95% CI for Adj OR	
	B	S.E	Wald	df	*P* value	Adjusted OR	Lower	Upper
Group 1						1		
Group 2	1.929	1.354	2.029	1	0.154	6.882	0.484	97.772
Age group (upto 40 years)						1		
Age group (> 40 years)	1.208	1.027	1.383	1	0.240	3.346	0.447	25.040
Males						1		
Females	-.979	.871	1.262	1	.261	.376	.068	2.072
1 st molar			5.824	2	.054			
2nd molar	−1.549	.955	2.632	1	.105	.212	.033	1.381
3rd molar	−3.843	1.672	5.285	1	.022	.021	.001	.567
Constant	.797	.455	3.064	1	0.080	2.219		

## Discussion

The prevalence of diabetic patients is steadily rising, as is the number of diabetics undergoing endodontic treatment. Nonetheless, a significant gap remains in research concerning the effectiveness of LA for this population. This is the first clinical trial that evaluated anesthetic success in confirmed diabetic patients. Results of the present study showed that IANB success was significantly higher in diabetic patients than non-diabetic patients. The null hypothesis for the study was thus rejected.

Our results contradict with a recently published National Dental Practice based Research Network Study by Weitz et al. [12], who investigated the association between diabetic status (among other preoperative factors) and anesthetic success in patients undergoing non-surgical RCT. Data from their study suggested that diabetic patients had almost 5 times higher chances of anesthesia failure than non-diabetic population. However, their method of reporting anesthetic success/failure was different from the current study, and patients were considered/labelled diabetic, based on their self-reported medical history. In addition, it was a retrospective study where patients were treated by multiple operators, which included a greater number of general dentists and fewer endodontists. Lastly, the type of local anaesthetics and pulpal diagnosis of teeth undergoing RCT were not specified. On the contrary, the present study was a prospective clinical trial where all recruited patients were confirmed diabetics with a specific diagnosis, were treated by a single operator (postgraduate student) using a standardised IANB and a patient-centred approach was adopted to determine anesthetic outcome.

Medical studies conducted in diabetic patients reported mixed findings regarding anesthetic success. While Gebhard et al. [16] reported higher success rate of supra-clavicular nerve block in diabetics, some other medical studies concluded that anesthetic success rate is not governed by diabetic status of the individual [17–19]. Gebhard et al. [16] speculated that nerve fibers in diabetics may be more sensitive to LA because of micro-vascular damage associated with diabetes or decreased sensation in their nerves (diabetic neuropathy) which makes anesthesia appear more successful. Levy and Lirk, based on a study conducted in diabetic rodents, stated that reasons for more effectiveness of LA in diabetics are both pharmacodynamic and pharmacokinetic [20, 21]. Pharmacodynamically, decreased inhibitory concentration of lidocaine was required to block global (voltage gated) sodium currents in dorsal root ganglion neurons from diabetic neuropathic rats compared to healthy rodents. From the pharmacokinetic perspective, the vascular supply and perfusion of diabetic neuropathic nerves was reduced making them more sensitive to local anesthetics, resulting in longer and denser blocks.

Weitz et al. [12] and colleagues conjectured that the lower anesthetic success rate in their study (compared to medical studies) may be due to the unique microvasculature of pulp and, unlike large peripheral nerves, pulp might respond to hyperglycemia differently. However, a recent study [8] has concluded that hyperglycemia may result in similar complications in the human dental pulp as in other body sites. These include denser connective tissue (fibrosis), reduced vascularity, pulpal calcifications, reduced potential for angiogenesis and healing, pro-inflammatory effects within pulp and degeneration of neural tissue [8]. Additionally, distinctive changes are observed in cells of diabetic patients [22]. For instance, excessive surplus electrons in mitochondria release more oxidants, which reduces mitochondrial action potential, leading to poor energy ATP synthesis and a decrease in the overall conduction potential of nerves. The peripheral nerve cells undergo glycosylation in diabetics which exert direct toxicity on the nerves along with endoneurial microangiopathy. All these effects, perhaps, translated clinically into decreased response or, in other words, enhanced anesthetic success in diabetics.

Age is a significant risk factor for developing type 2 DM [23], with the disease being more prevalent among individuals aged 45 to 64 years [24]. The present study observed a similar trend, noting that the majority of diabetic patients were older compared to their non-diabetic counterparts. Interestingly, elderly diabetic patients experienced higher anesthetic success than younger non-diabetic patients, suggesting that age could act as a confounding factor. A more effective study design i.e. matched case–control design or stratified sampling might have reduced confounding (especially age). To adjust for age, logistic regression was performed in our study which revealed that age had no significant effect on IANB success. Nevertheless, the exact role age plays in determining anesthetic success remains a debatable topic. Some endodontic studies [25–27] indicate that older individuals may experience less pain or require less supplemental anesthesia than younger patients, while other research has found no substantial effect of age on intra-operative pain [28]. A clinical study [29] compared pulp sensibility responses to cold and electric stimuli in healthy and type 2 diabetic patients and found that in elderly diabetic patients (> 45 years), the response to the cold test was significantly reduced, particularly in maxillary premolars. In addition, age related pulpal structural changes could have affected the anesthesia success but literature on age associated IANB success is scarce. Further studies are necessary to establish a definitive clinical correlation between age and the pain experienced by patients during RCT.

As per the Centres for Disease Control and Prevention (CDC) data, 8.5 million people (23% of adults) in the US have undiagnosed diabetes [30]. In developing countries like India, more than 57% of diabetic individuals remain undiagnosed [31]. So, all patients were tested for HbA1c levels rather than relying on self-reported status. Interestingly, three patients who presented as non-diabetic turned out to be diabetic based on higher HbA1c levels and physician consultation.

Diabetes can be diagnosed by fasting plasma glucose value, 2 h plasma glucose value during a 75-g oral glucose tolerance test or HbA1c levels [2]. We used HbA1c values as it is convenient (fasting not required) and is not affected by stress, changes in diet, or illness [2]. The tests for HbA1c were standardized and performed in a certified laboratory. Our study excluded prediabetics (HbA1c 5.7—6.4) since they did not clearly distinguish between diabetics and non-diabetics. There is no general recommendation by the American Association of Endodontists, ADA or any other international dental association for the glucose level up to which RCT can be safely administered. Elective dental treatment in poorly controlled diabetics is delayed until the condition is stable [32]. The Society for Ambulatory Anesthesia Consensus Statement recommends a perioperative blood level of less than 180 mg/dl for ambulatory settings [33, 34]. Thus, we set a cut-off value HbA1c > 8 (which corresponds to 183 mg/dl) and patients with HbA1c > 8 were excluded. Excluding poorly controlled diabetics (HbA1c > 8) limits generalizability to typical diabetic populations. To prevent confounding effects, diabetic patients with other comorbidities such as cardiovascular complications, end-stage renal disease, hypertension, depression, thyroid gland diseases and chronic obstructive pulmonary disease, were excluded from the study [35]. This led to the exclusion of many diabetics, and therefore, the current study’s sample might not truly represent most diabetics.

In the present study, we also included diabetic patients who were taking oral hypoglycemic drugs. Hyperglycemic patients are routinely prescribed oral hypoglycemics like biguanides, meglitinides, thiazolidinedione derivatives and DPP-4 inhibitors. Thiazolidinedione derivatives such as pioglitazone and rosiglitazone, Bisguanides like metformin and DPP-4 inhibitors like sitagliptin and linagliptin possess anti-inflammatory potential [36, 37]. These drugs exert their anti inflammatory effect via reducing the production of inflammatory markers like C-reactive proteins, interleukin 6 and tumour necrosis factor-α [36], which might have influenced study results. However, the current study did not consider this effect, which may be a limitation. Moreover, diabetic neuropathy may reduce subjective pain reporting, thus potentially inflating perceived anesthetic success.

In mandibular molars with SIP, achieving successful LA is challenging. To increase anesthetic success, plethora of endodontic studies have employed many anesthetics such as articaine, prilocaine, mepivacaine [38, 39] and other supplemental injection techniques like infiltration, intraligamentary or intraosseous injections. In our study, however, after a single successful IANB, patients who reported moderate to severe pain during endodontic intervention were included in failures. Though it was done to evaluate IANB success exclusively, it may not reflect clinical scenarios where supplemental injections are routinely administered during RCT. In addition, our study was a single centre trial, had a small sample size and has limited generalizability due to exclusion of insulin dependent diabetics. Also, we did not measure the post-procedure pain levels, which would have been more insightful.

Future multicentric studies with large sample size, minimal confounding variables, use of different local anaesthetics, including broad diabetic sample (insulin dependent diabetics, patients with co-morbidities), different pulpal and periapical diagnoses and ascertaining the effect of oral hypoglycemic drugs on LA outcome, would help us better understand the impact of diabetes mellitus on the LA in dental patients.

## Conclusion

Within the limitations of the present study, IANB is more successful in type 2 diabetic patients compared to non-diabetics. However, these conclusions are preliminary and must be interpreted cautiously due to confounding variables, especially age.

## Data Availability

Data are available from the corresponding author upon reasonable request.

## Abbreviations

IANB: Inferior Alveolar Nerve Block
SIP: Symptomatic Irreversible Pulpitis
DM: Diabetes Mellitus
